# Gut dysbiosis is prevailing in Sjögren’s syndrome and is related to dry eye severity

**DOI:** 10.1371/journal.pone.0229029

**Published:** 2020-02-14

**Authors:** Jayoon Moon, Se Hyun Choi, Chang Ho Yoon, Mee Kum Kim

**Affiliations:** 1 Department of Ophthalmology, Seoul National University College of Medicine, Seoul, Republic of Korea; 2 Laboratory of Ocular Regenerative Medicine and Immunology, Seoul Artificial Eye Center, Seoul National University Hospital Biomedical Research Institute, Seoul, Republic of Korea; 3 Department of Ophthalmology, Hallym University Sacred Heart Hospital, Anyang-si, Gyeonggi-do, Republic of Korea; University of Bergen, NORWAY

## Abstract

**Objective:**

To investigate gut dysbiosis in patients with Sjögren’s syndrome (SS) or dry eye syndrome (DES) compared to normal subjects and to evaluate the association of dysbiosis with dry eye severity.

**Methods:**

10 subjects with SS, 14 subjects with DES and 12 controls were enrolled. Corneal staining, tear break up time (TBUT) and tear secretion were evaluated. Bacterial genomic 16s rRNA from stool samples were analyzed. Main outcomes were microbiome compositional differences among groups and their correlation to dry eye signs.

**Results:**

Gut microbiome analysis revealed significant compositional differences in SS compared to controls and DES. In phylum, Bacteriodetes increased, while Firmicutes/Bacteroidetes ratio and Actinobacteria decreased (p<0.05). In genus, Bifidobacterium was reduced (vs controls; p = 0.025, vs DES; p = 0.026). Beta diversity of SS also showed significant distances from controls and DES (p = 0.007 and 0.019, respectively). SS showed decreased genus of Blautia (p = 0.041), Dorea (p = 0.025) and Agathobacter (p = 0.035) compared to controls and increased genus of Prevotella (p = 0.026), Odoribacter (p = 0.028) and Alistipes (p = 0.46) compared to DES. On the other hand, DES only had increased genus Veillonella (p = 0.045) and reduced Subdoligranulum (p = 0.035) compared to controls. Bacteroidetes, Actinobacteria and Bifidobacterium were significantly related with dry eye signs (p<0.05). After adjustment of age, gender and group classification, multivariate linear regression analysis revealed tear secretion was strongly affected by Prevotella (p = 0.025). With additional adjustment of hydroxychloroquine use, TBUT was markedly affected by Prevotella (p = 0.037) and Actinobacteria (p = 0.001).

**Conclusions:**

Sjögren’s syndrome showed significant gut dysbiosis compared to controls and environmental dry eye syndrome, while dry eye patients showed compositional changes of gut microbiome somewhere in between Sjögren’s syndrome and controls. Dysbiosis of the gut microbiota was partly correlated to dry eye severity.

## Introduction

Sjögren’s syndrome (SS) is one of the most common chronic autoimmune diseases and is associated with lymphocytic infiltration in exocrine glands and various other organs. Its symptoms include dry mouth, musculoskeletal pain and dry eyes[1, 2]. Among these symptoms, dry eye is one of the most discomforting symptoms that SS patients complain about and SS-related dry eye treatment is challenging compared to environmental dry eye syndrome (DES)[3, 4]. Although, treatment of dry eye is currently standardized[5, 6], there are still unmet needs for SS patients given their disease’s more symptomatic severity.

Ever since the first completion of a whole genome of a free-living organism in 1995, numerous studies on microbes and viruses for genome sequencing have been conducted and are still in progress with further investigations of their relation to human health and diseases[7–10]. Recently, several studies have focused on how the gut microbiota influence health and diseases and had promising results concerning autoimmune diseases, such as Crohn’s disease, systemic lupus erythematosus (SLE) and autoimmune uveitis[11–16]. Intestinal epithelial cells are reported to play an essential role in balancing gut microbiota and their metabolic changes can result in dysbiosis in certain autoimmune diseases[10, 17]. The gut microbiota has been reported to be interactive in maintaining balance in immune responses between regulatory T cells (Tregs) and T helper 17 (Th17) cells at mucosal surface and to act as a trigger of inducing autoimmunity, such as SLE, rheumatic diseases or SS[9, 18].

Currently, not only ocular microbiome but also gut microbiome have received much attention for intervening ocular homeostasis and diseases such as dry eye, uveitis, age related maculopathy, and diabetic retinopathy etc[19]. Dysbiotic gut microbiome is also reported to be associated with ocular mucosal diseases in SS[20]. However, there are only few reports regarding the dysbiosis of the gut in Sjögren’s syndrome[20, 21]. Given that food habits and ethnicity may affect the gut microbiota, investigation of the gut microbiota of SS patients should be further conducted in different regions including Asia. In addition, our previous study using Sjögren’s syndrome animal model reported beneficial effects of probiotics on dry eye[22]. Therefore, we hypothesized that Sjögren’s syndrome patients in Korea may have gut dysbiosis. If so, beneficial effects of probiotics on dry eyes for Sjögren’s syndrome in a clinical trial could be investigated based on that clinical evidence.

Herein, the main objectives of this study were to investigate compositional differences of gut microbiome in patients with SS or DES compared to normal subjects and to evaluate their correlations to dry eye severity.

## Materials and methods

### Subjects

This prospective study was adhered to the ethical standards of Declaration of Helsinki and was approved by the Institutional Review Board of Seoul National University College of Medicine (IRB number: 1711-138-902, Seoul, South Korea). Written informed consent was obtained from all participants.

A total of 36 subjects, 12 of whom were healthy volunteers, who had visited our Corneal clinic (M.K.K.) of Seoul National University Hospital (Seoul, South Korea) between June 30^th^, 2018 and June 30th, 2019, were enrolled. 36 subjects consisted of 10 subjects with confirmed Sjögren’s syndrome (SS), 14 subjects with environmental dry eye syndrome (DES) and 12 healthy volunteers. SS group included the subjects who had been diagnosed with primary SS according to the 2016 ACR/EULAR criteria by an internal medicine physician[23]. Secondary SS subjects were excluded from the SS group. DES group included subjects complaining of dry eye symptoms and less than 10 seconds of tear break up time (TBUT) which was defined as environmental dry eye syndrome by the Korean Guidelines[24]. DES group was also confirmed to have no existing auto-antibodies or rheumatology-related diseases. Healthy volunteers without dry eye symptoms or longer than 10 seconds of TBUT were included as the control group.

Any subject who reported intake of any kind of probiotics or oral antibiotics within a week prior to enrollment was excluded. Among the groups, there were no statistically significant demographic differences in age and gender, and all subjects were Korean (Table 1). 5 subjects (50%) in SS group had been taking oral hydroxychloroquine (Table 1).

**Table 1:** General demographics of all subjects in each group.

	SS (n = 10)	DES (n = 14)	Controls (n = 12)	p value
**Age (years)**	58.50 ± 3.05 (47–75)	46.29 ± 2.60 (24–63)	47.50 ± 4.05 (22–62)	0.061*
**Gender** **(Male: Female)**	0 (0%): 10 (100%)	2 (17.3%): 12 (85.7%)	3 (25%): 9 (75%)	0.250^**§**^
**Hydroxychloroquine medication**	5 (50%)	0	0	0.0006^**§**^

SS: Sjogren’s syndrome group, DES: dry eye syndrome group

*Analysis of variance

^**§**^Kruskal-Wallis test

### Study design

In this prospective case-control study, dry eye signs of corneal staining evaluation with National Eye Institute (NEI) score, TBUT, and Schirmer test were measured at all subjects’ first visits. All subjects were given stool sample kits for collection at home. Stool samples were collected and preserved at -80°C until analysis. Bacterial genomic DNA was extracted from collected stool samples for analysis of microbial composition comparison among groups (taxonomic relative abundance was compared), alpha diversity (species richness by Chao1 indices and Shannon diversity index) and beta diversity (Unifrac Principal coordinates analysis (PCoA) at genus level). Permutational multivariate analysis of variance (PERMANOVA) was used to find significance in Unifrac PCoA. Compositional abundance differences were first identified from each sample by analysis using linear discriminant analysis (LDA) of effect size (LEfSe) and the Kruskal-Wallis test[25]. Univariate linear regression analysis between dry eye signs and clinically important microbials with significant differences among groups was performed. Multivariate regression analysis was performed using the stepwise selection method to identify independent significant microbials affecting dry eye signs after adjustment of potential confounders such as age, gender, group classification and use of hydroxychloroquine. The average values of NEI scoring, TBUT, and Schirmer test results from both eyes were used for these analyses.

### Dry eye sign analysis

All subjects underwent slit lamp microscopic examinations for evaluation of corneal staining using NEI scores[26] and TBUT[27]. Corneal staining was evaluated using Fluorescein® (Haag-Streit International, Koniz, Switzerland) paper strips after placing one drop of sterile saline. NEI scores for evaluation of corneal staining assesses 5 areas of the cornea on a 0–3 scale with a total score generated by summation of the 5 section scores (0–15)[26]. TBUT, using the same Fluorescein® paper strips used for corneal staining evaluation, was measured three times and averaged in each eye. Basal and reflex tear secretion was measured in millimeters at 5 minutes using Schirmer’s strips without prior instillation of topical anesthesia[28].

### Fecal microbiota analysis

All collected feces were stored at -80°C and was referred to Chunlab, Inc. (Seoul, Korea) for analysis. Fecal microbiota analysis was done by polymerase chain reaction (PCR) amplification, which was performed using extracted DNA and bacterial PCR primers 341F (5’-TCGTCGGCAGCGTC-AGATGTGTATAAGAGACAG-CCTACGGGNGGCWGCAG-3’; underlining sequence indicates the target region primer) and 805R (5’-GTCTCGTGGGCTCGG-AGATGTGTATAAGAGACAG-GACTACHVGGGTATCTAATCC-3’) targeting V3-V4 regions of 16S rRNA gene. The reaction conditions were as follows: 3 minutes of initial denaturation at 95°C, 25 cycles of 30 seconds’ denaturation at 95°C, 30 s primer annealing at 55°C, 30 s elongation at 72°C, and final extension at 72°C for 5 minutes. Secondary amplification was performed using i5 forward primer (5’-AATGATACGGCGACCACCGAGATCTACAC-XXXXXXXX-TCGTCGGCAGCGTC-3’; X indicates the barcode region) and i7 reverse primer (5’-CAAGCAGAAGACGGCATACGAGAT-XXXXXXXX-GTCTCGTGGGCTCGG-3’) for attaching the Illumina NexTera barcode. The second amplification condition was identical to that of the first amplification except for the amplification cycle setting was to 8. The amplified products were confirmed using gel electrophoresis on 1.0% agarose gel and visualized under a Gel Doc system (BioRad, Hercules, CA, USA). The product size and quality were evaluated on a Bioanalyzer 2100 (Agilent, Palo Alto, CA, USA) using a DNA 7500 chip. Mixed amplicons were pooled and sequenced using an Illumina MiSeq Sequencing system (Illumina, Inc., San Diego, CA, USA) according to the manufacturer’s instruction at Chunlab, Inc. (Seoul, Korea). After chimera check, the EzBioCloud database (http://ezbiocloud.netwas used for taxonomic classification. To detect chimera on reads that contain <97% best hit similarity rate, UCHIME and the non-chimeric 16S rRNA database from EzBioCloud were used.

Clustering of the sequences and grouping according to operational taxonomic units (OTU) were done. The Bioplug software (Chunlab, Seoul, Korea) was used to summarize the OTU data and to calculate microbial alpha and beta diversity for each sample. Alpha diversity is an analysis of the diversity within a community, including the Chao1 index and Shannon index. Rank sum test analysis was used to analyze significant differences regarding alpha diversity. LDALEfSe, which was performed using the LEfSe tool provided in the public domain(http://huttenhower.sph.harvard.edu/lefse/), and the Kruskal-Wallis test, which was performed by using the Wilcox test function of the stats R package, were used to estimate the effect of abundance of each sample on the effect of differences, and to identify the bacterial taxa that shows significant differences in their demarcation. Only those taxa that showed a p-value < 0.05 and log LDA score ≥2 were ultimately considered. Multiple test corrections were based on the FDR. FDR of 0.05 was used as a statistically significant cutoff.

### Statistical analysis

Statistical analyses were performed using SPSS software version 22 (SPSS, Inc, Chicago, IL) and GraphPad software version 6.01 (GraphPad Software, San Diego, CA). For dry eye signs and demographic comparison among groups, analysis of variance (ANOVA) and Kruskal-Wallis tests were performed. Comparison of gut microbiome composition among groups was done using Wilcoxon rank-sum test. P values less than 0.05 were accepted as statistically significant. The results are presented as mean ± standard error of the mean (SEM) unless otherwise indicated.

## Results

**Fig 1:**
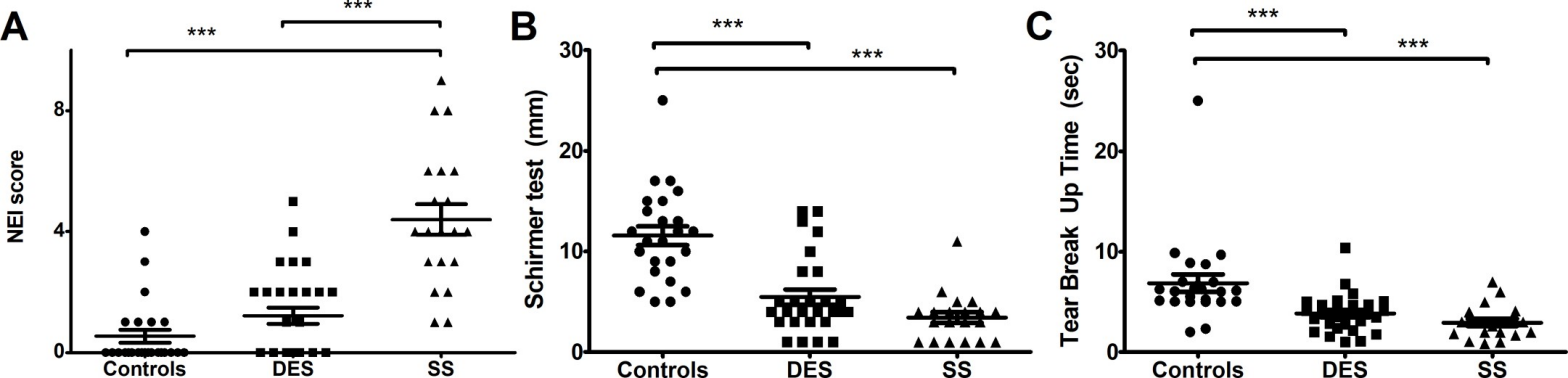
Dry eye indices in controls, DES, and SS groups. NEI score was significantly higher in SS than in both controls and DES (p < 0.0001, A). Tear secretion was lower in both SS and DES than in controls (p < 0.0001, B) and tear break up time was shorter in both SS and DES than in controls (p < 0.0001, C). DES: dry eye syndrome group, SS: Sjogren’s syndrome group, NEI: National Eye Institute, mm: millimeters, sec: seconds ***p < 0.0001, Analysis of variance.

First, we evaluated severity of dry eye in each group. Clinically, SS group showed significantly higher NEI scores, lower tear secretion and shorter TBUT compared to controls (p < 0.0001, Fig 1). Aside from NEI scores (p < 0.0001 vs DES, Fig 1A), TBUT and tear secretion in SS group were not different from those in DES (p > 0.05; Fig 1B and 1C). DES also showed lower tear secretion and shorter TBUT compared to controls (p < 0.0001, Fig 1B and 1C).

**Fig 2:**
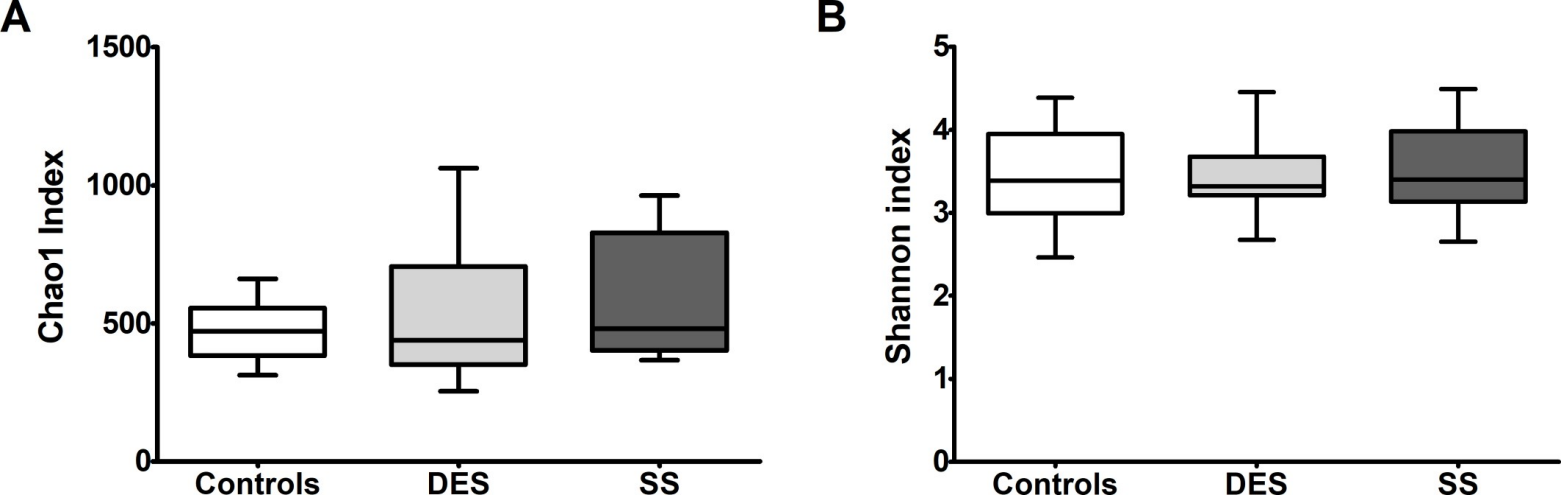
Species richness and Shannon diversity analysis of Controls, DES and SS groups. Species richness analyzed by Chao 1 did not show any differences among groups (controls vs. DES; p = 0.877, controls vs. SS; p = 0.553, SS vs. DES; p = 0.447, Wilcoxon rank-sum test) (A). Shannon diversity index also did not reveal any differences among groups (controls vs. DES; p = 0.918, controls vs. SS; p = 0.792, SS vs. DES; p = 0.907, Wilcoxon rank-sum test) (B). DES: dry eye syndrome group, SS: Sjogren’s syndrome group Bars indicate maximum and minimum values.

Next, we analyzed alpha diversity of each groups’ gut microbiome. Chao 1 index describing species richness revealed no significant differences (controls vs. DES; p = 0.877, controls vs. SS; p = 0.553, SS vs. DES; p = 0.447, Fig 2A). Also, Shannon diversity index did not reveal significant differences among all groups (controls vs. DES; p = 0.918, controls vs. SS; p = 0.792, SS vs. DES; p = 0.907, Fig 2B).

**Fig 3:**
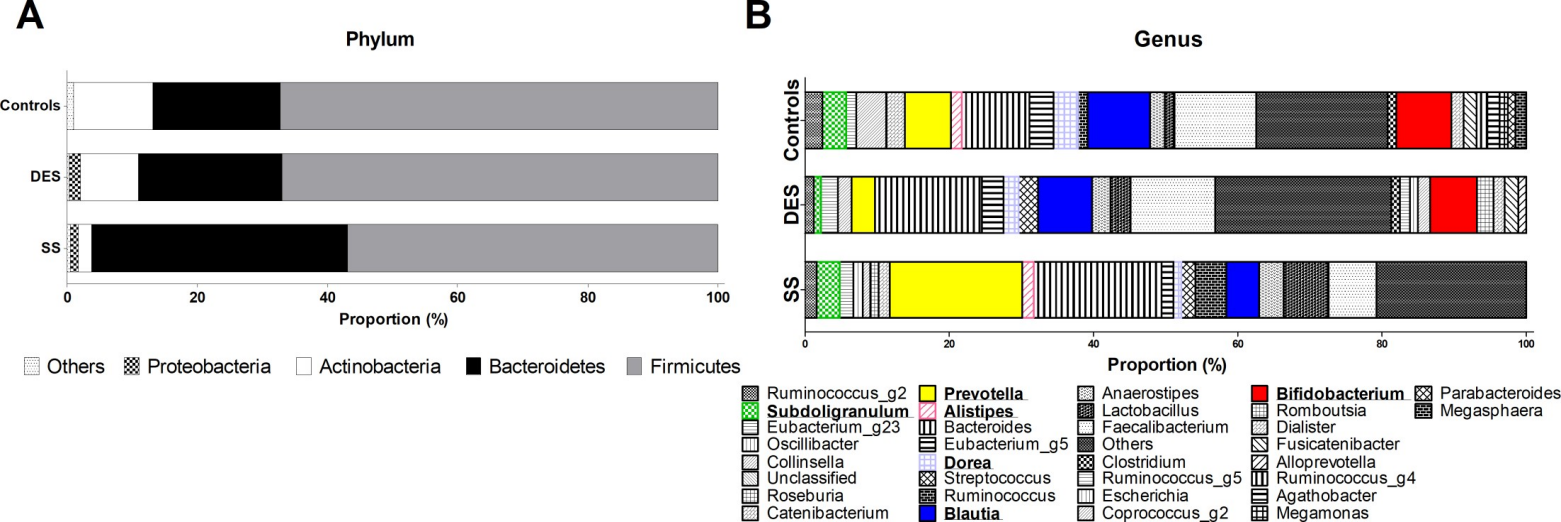
Taxonomic proportions according to compositions in phylum and genus of Controls, DES, and SS groups. Overall, taxonomic relative abundance in phylum (A) and genus (B) is shown for all groups. DES: dry eye syndrome group, SS: Sjogren’s syndrome group.

**Fig 4:**
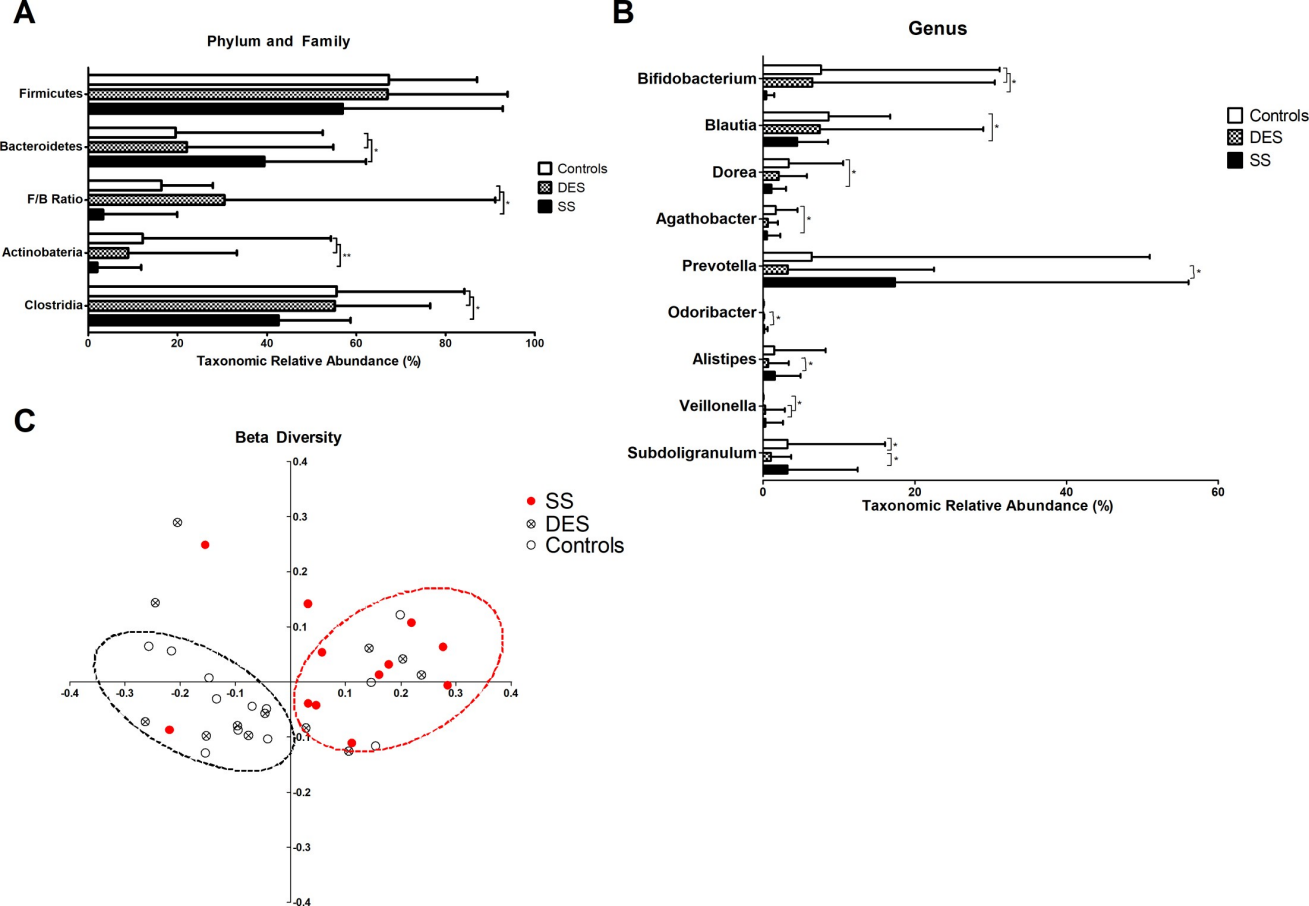
Taxonomic relative abundance according to Phylum, Family and Genus, and principle coordinates analysis of Controls, DES, and SS groups. In phylum, significantly abundant Bacteroidetes (p < 0.05) and reduced Actinobacteria (p < 0.01) were seen in SS compared to both controls and DES (A). Family Clostridia was also significantly reduced in SS compared to both controls and DES (p < 0.05) (A). In genus, SS had decreased Bifidobacterium (vs controls; p = 0.025, vs DES; p = 0.026) (B). Genus of Blautia (p = 0.041), Dorea (p = 0.025) and Agathobacter (p = 0.035) were also reduced in SS compared to controls. Increased genus of Prevotella (p = 0.026), Odoribacter (p = 0.028) and Alistipes (p = 0.046) were observed in SS compared to DES (B). Genus Veillonella was higher in SS (p = 0.045) and DES (p = 0.045) than in controls. Genus Subdoligranulum was lower in DES than in controls and SS (p = 0.035 and 0.026, respectively) (B). Beta diversity of genus analyzed by Unifrac principal coordinates analysis revealed SS to have significant distances from DES and controls (PERMANOVA, p = 0.019 and 0.007, respectively) (C). No significant distance was observed between DES and controls (PERMANOVA, p = 1.000) (C). DES: dry eye syndrome group, SS: Sjogren’s syndrome group, PERMANOVA: permutational multivariate analysis of variance Bars in A and B indicate maximum value. *p<0.05, Wilcoxon rank-sum test, **p<0.01, Wilcoxon rank-sum test.

Thereafter, we looked at compositional differences of gut microbiome at the level of phylum, family and genus of each group (Fig 3). SS showed marked differences in phylum of the following compared to controls and DES (Fig 4A); Increased Bacteriodetes (p = 0.015 and 0.040, respectively), decreased Firmicutes to Bacteroidetes (Firmicutes/Bacteroidetes) ratio (p = 0.015 and 0.035, respectively) and Actinobacteria (p = 0.005 and 0.006, respectively). Family Clostridia was also reduced in SS (vs controls; p = 0.012, vs DES; p = 0.008; Fig 4A). In genus, SS had decreased Bifidobacterium (vs controls; p = 0.025, vs DES; p = 0.026; Fig 4B). Also, SS revealed reduced genus of Blautia (p = 0.041), Dorea (p = 0.025) and Agathobacter (p = 0.035) compared to controls and increased genus of Prevotella (p = 0.026), Odoribacter (p = 0.028) and Alistipes (p = 0.046) compared to DES (Fig 4B). Genus Veillonella was more abundant in SS (p = 0.045) and DES (p = 0.045) than controls. Genus Subdoligranulum was reduced in DES than in controls and SS (p = 0.035 and 0.026, respectively; Fig 4B).

Then, we evaluated beta diversity of each groups’ gut microbiome. Unifrac PCoA revealed significant differences in SS compared to controls and DES (p = 0.007 and 0.019, PERMANOVA, respectively, Fig 4C). While there was no difference in distance between DES and controls (p = 1.000, PERMANOVA, Fig 4C).

**Fig 5:**
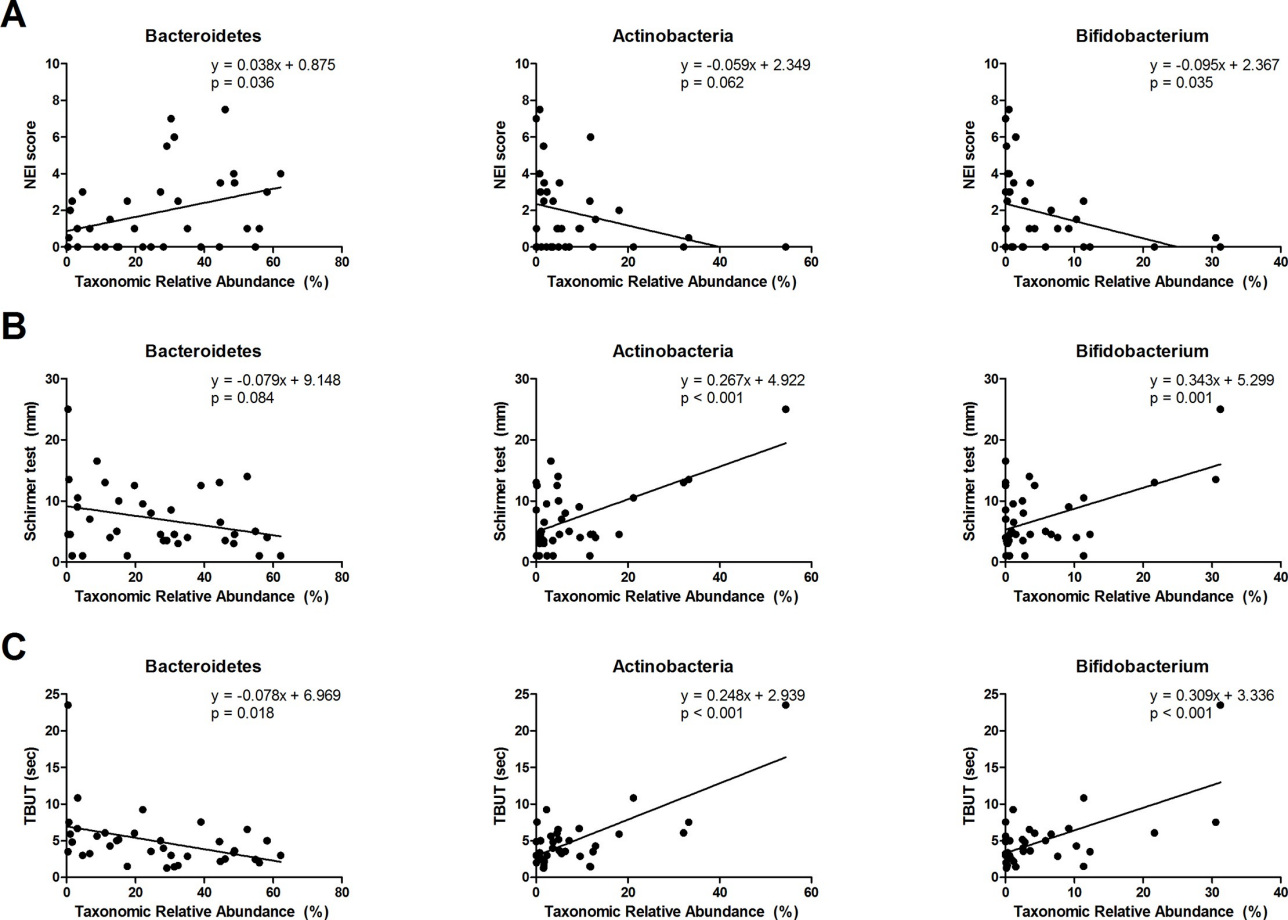
Univariate linear regression analysis between dry eye indices and intestinal microbiome. NEI score showed significant positive relation with Bacteroidetes (p = 0.036, R^2^ = 0.123) and negative relation with Bifidobacterium (p = 0.035, R^2^ = 0.124), while there was no significance with Actinobacteria (p = 0.062, R^2^ = 0.099) (A). Tear secretion showed positive relation with both Actinobacteria (p < 0.001, R^2^ = 0.331) and Bifidobacterium (p = 0.001, R^2^ = 0.263), while there was no significance with Bacteroidetes (p = 0.084, R^2^ = 0.085) (B). TBUT showed negative relation with Bacteroidetes (p = 0.018, R^2^ = 0.154), and positive relation with both Actinobacteria (p < 0.001, R^2^ = 0.534) and Bifidobacterium (p < 0.001, R^2^ = 0.401) (C). DES: dry eye syndrome group, SS: Sjogren’s syndrome group, NEI: National Eye Institute, TBUT: Tear break up time, mm: millimeters, sec: seconds.

Finally, we investigated relationship between dysbiosis and dry eye indices (Fig 5). Univariate linear regression analysis showed that NEI score had positive relation with Bacteroidetes (p = 0.036, R^2^ = 0.123) and negative relation with Bifidobacterium (p = 0.035, R^2^ = 0.124), while there were no significant relation with Actinobacteria (p = 0.062, R^2^ = 0.099) (Fig 5A). There was significant positive relation between tear secretion and Actinobacteria (p < 0.001, R^2^ = 0.331) and Bifidobacteria (p = 0.001, R^2^ = 0.263), while there was no significance with Bacteroidetes (p = 0.084, R^2^ = 0.085) (Fig 5B). TBUT revealed to have negative relation with Bacteroidetes (p = 0.018, R^2^ = 0.154), and strong positive relation with both Actinobacteria (p < 0.001, R^2^ = 0.534) and Bifidobacteria (p < 0.001, R^2^ = 0.401) (Fig 5C). Meanwhile, Prevotella and Blautia did not show significant relation with dry eye indices (NEI and Prevotella p = 0.192, R^2^ = 0.049; NEI and Blautia p = 0.140, R^2^ = 0.063; Schirmer test and Prevotella p = 0.881, R^2^ = 0.001; Schirmer test and Blautia p = 0.139, R^2^ = 0.063; TBUT and Prevotella p = 0.546, R^2^ = 0.011; TBUT and Blautia p = 0.064, R^2^ = 0.097). After adjustment of age, gender and group classification, multivariate linear regression analysis revealed that tear secretion was only significantly affected by Prevotella (β = 0.264, p = 0.025). Also, TBUT showed to be only significantly influenced by Actinobacteria (β = 0.500, p = 0.007). With additional adjustment of the use of hydroxychloroquine, along with age, gender and group classification, TBUT showed to be markedly affected by both Prevotella (β = 0.254, p = 0.037) and Actinobacteria (β = 0.658, p = 0.001), while the significant correlation seen between tear secretion and Prevotella remained. However, NEI score proved to not be affected by gut dysbiosis.

## Discussion

Our study demonstrates that gut dysbiosis is prevalent in Sjögren’s syndrome and gut dysbiosis is associated with ocular disease severity. Also, our results showed that environmental dry eye syndrome lies somewhere in between Sjögren’s syndrome and healthy groups. It also presents dysbiosis of the gut microbiota is partly correlated to dry eye severity.

Gut microbiota and the immune system are physiologically interactive during developmental process and gut microbiota has been reported to play a crucial role in human diseases such as chronic inflammatory diseases and metabolic diseases[10, 29]. Emerging evidences indicate that gut dysbiosis contributes to the pathophysiology or exacerbation of autoimmune diseases, including rheumatoid arthritis, SLE, systemic sclerosis, ankylosing spondylitis, and Sjögren’s syndrome through the imbalance of the immune system[18]. Dysbiotic patterns vary depending on the disease. In the field of ophthalmology, the gut-eye axis has been actively investigated in autoimmune uveitis, and autoimmune dry eye[11, 13, 19, 20]. A recent prospective human study showed that supplementation with Akkermansia muciniphila intervenes with metabolic diseases[30]. Therefore, understanding the dysbiotic patterns of gut microbiota in each autoimmune disease may shed a light on customization of probiotic therapy in each disease. Many mouse models regarding autoimmune diseases have shown variable expressions of gut dysbiosis[31, 32]. However, different genetic backgrounds largely affect the composition of gut microbiota. Therefore, achieving information about the composition of gut microbiota in humans with a specific disease would be clinically relevant for the development of precision medicine.

Imbalance of gut microbiota affect the imbalance of T helper 1 (Th1) and Th17 cells, polarization of Tregs, increase in permeability of intestinal epithelial cells, and production of short-chain fatty acids (SCFAs) etc[18]. Increase in Bacteroides fragilis can stimulate Th1-mediated immune responses. The gut microbiota is consisted of the main two phyla, Bacteroidetes and Firmicutes, and the rest composed of bacteria such as Proteobacteria, Actinobacteria, and Fusobacteria. Among these phyla, Firmicutes/Bacteroidetes ratio shift is known to be the first indication for gut dysbiosis. In chronic inflammatory diseases like SLE and systemic sclerosis, elevated Bacteroidetes levels and a reduced Firmicutes/Bacteroidetes ratio have been observed. In our study, gut dysbiosis were only noted in Sjögren’s syndrome while patients with environmental dry eye syndrome maintained similar gut microbiota compositions with those of healthy volunteers. Although, studies for gut microbiota in Sjögren’s syndrome have not been conducted much so far, this dysbiosis can be supported by previous studies[20, 21]. One study reported that gut dysbiosis was prevalent in Sjögren’s syndrome and was associated with systemic severity, but they did not mention how the composition of the gut microbiota changed[21]. In another study, gut dysbiosis in Sjögren’s syndrome was observed and they reported reduced abundance of Bacteroides, Parabacteroides, Faecalibacterium, and Prevotella, while there were increased abundance of Pseudobutyrivibrio, Escherichia/Shigella, Blautia, and Streptococcus[20]. Our data presented with increased Bacteroidetes levels and decreased Firmicutes/Bacteroidetes ratio like SLE or systemic sclerosis. In addition, level of Actinobateria and Clostridia were significantly decreased. The Actinobacteria phylum, which includes Bifidobacterium and Collinsella genera, is known to have beneficial effects on intestinal health by adjusting bile acids and regulating virulence of enteric pathogens[33]. Notably, level of Bifidobacterium genus was remarkably lower in Sjögren’s syndrome than in environmental dry eye syndrome and healthy volunteers. Blautia, Dorea, and Agathobacter genera were also reduced in Sjögren’s syndrome than in healthy volunteers. Given that Bifidobacterium and Lactobacillus are typically reduced in chronic inflammatory states[18] and are involved with intestinal health, our study results correspond well with previous studies[21] and may be related to the pathogenesis of Sjögren’s syndrome. In consideration of Prevotella, a crucial genus in the onset of rheumatoid arthritis, increased abundance in our study may also be involved with the inflammatory pathogenesis of Sjögren’s syndrome. This finding was different from a previous study that reported decrease of Prevotella genus in Sjögren’s syndrome[20]. This can be explained by different dietary habits in Koreans who consume more vegetables than meat which in turn may affect the composition of the gut microbiota that can differ from the western population[34] and patient’s preference for vegetable diets under poor general medical conditions. For instance, fiber-rich diet has been reported to be associated with increasing Prevotella in the intestine[16, 35]. Also, Dorea was reported to increase in type 2 diabetes after intake of diet rich in carbohydrates, whole-grain and vegetables[16].

Among the SCFAs, butyrate is a crucial metabolite providing energy for colonic epithelial cells to maintain intestinal barrier functions and having anti-inflammatory functions. Butyrates are produced by interactions between Bifidobacterium and butyrate-producing colon bacteria that mostly belongs to the Firmicutes phylum, such as Faecalibacterium prausnitzii and Eubacterium rectaleas, which are one of the major role players[36, 37]. Another important butyrate-producing bacterial species are known to be Roseburia spp., (Roseburia faecis, Roseburia inulinivorans, Roseburia gutis, and Roseburia hominis), Eubacterium spp. (Eubacterium hallii), Anaerostipes spp. (Anaerostipes butyraticus, Anaerostipes caccae, and Anaerostipes hadrus), and Butyricicoccus pullicaecorumin in the human colon[36]. In our study, there was reduction of Eubacterium hallii in Sjögren’s syndrome compared to controls (p = 0.041) and environmental dry eye syndrome (p = 0.089), while other butyrate-producing bacterial species showed no significant differences. This finding may be evidence of Sjögren’s syndrome’s possible association with butyrate-related immune dysregulation and disruptive gut barrier function.

Veillonella group, a genus of potential pathobionts, has been reported to be strongly associated with autoimmune hepatitis[38]. In our study, we have found significantly more abundant Veillonella in both environmental dry eye syndrome and Sjögren’s syndrome than in healthy volunteers. This finding may be evidence to support gut dysbiosis’ relation in disrupting gut barrier function and leading to ocular surface inflammation. Also, Odoribacter group has been reported to decrease in SLE compared to healthy controls[39]. However, in our study, Sjögren’s syndrome revealed to have increased abundance of Odoribacter compared to environmental dry eye syndrome. Considering that only a very small portion of Odoribacter was found in all groups, its significance seems to not be clinically relevant. Nevertheless, further investigations of gut microbials with even the lowest proportions and their possible influence on gut dysbiosis and autoimmune diseases are necessary to fully understand the association between gut dysbiosis and human diseases.

Regarding alpha diversity, there were no differences among all groups in our study. This was different from that in a previous study[20]. Different diet habits or different severity of Sjögren’s syndrome included may be the possible causes.

Of note, our study revealed that dry eye severity was closely related to dysbiotic composition of various bacteria. Especially, reduced level of Bifidobacterium genus was significantly related to low tear secretion, short TBUT, and high corneal staining score which indicate severe ocular surface disease. Decreased level of Bacteroidetes and Actinobacteria phyla were also involved in some of the dry eye indices. Interestingly, however, after adjustment of confounding factors, multivariate regression analysis resulted in unexpected results. Prevotella genus was revealed to significantly affect both tear secretion and tear film stability when it did not have any significance in univariate linear regression analysis. This insignificance seen prior to multivariate analysis may be due to confounding factors, such as age, gender, group classifications and use of hydroxychloroquine. Also, Actinobacteria phylum was seen to significantly affect TBUT which was also seen in univariate linear regression analysis. Therefore, we can assume that reduced abundance of Actinobacteria and increased Prevotella have a strong association and influence on tear film stability. These findings suggest that gut dysbiosis in association with or without other confounding factors, such as age, gender etc., have clinically important influences on the severity of dry eye. At the best of our knowledge, it is a noble finding to be reported first.

In fact, our previous study using a Sjögren’s syndrome animal model presented that probiotics therapy with 5 compositions of Lactobacillus casei, Lactobacillus acidophilus, Lactobacillus reuteri, Bifidobacterium bifidum, and Streptococcus thermophilus alleviated ocular surface disease[22]. The fact that Bifidobacterium genus was decreased in Sjögren’s syndrome may indicate possible feasibility of specific probiotics therapy in these patients with severe dry eye signs.

Regarding patients with environmental dry eye syndrome, the compositional changes of gut microbiota was somewhere between those of Sjögren’s syndrome and controls. It suggests that pathogenesis of environmental dry eye syndrome would be different from the immune-dysregulation in Sjögren’s syndrome[40], although many studies revealed the culprit role of Th17 cells in environmental dry eyes[41–43]. Interestingly, Subdoligranulum genus was significantly decreased in environmental dry eye syndrome compared to controls and Sjögren’s syndrome. Reduced abundance of Subdoligranulum genus has been reported to be associated with lower score of Healthy Eating Index (HEI)[44] and negatively correlated with Behcet syndrome[45]. Therefore, the role of Subdoligranulum genus is not clear in autoimmune diseases and requires further investigation. As the environmental dry eye syndrome included in this study had mild dry eye signs defined by short TBUT (<10 sec), the changes of the gut microbiota may be minimal in this study. For that reason, further investigations regarding the gut dysbiosis in environmental dry eye syndrome with severe dry eye signs should be considered.

This study is limited by the small study size and the fact that the collected feces may not be a perfect representative of each group and their compositions may be different from the composition obtained from gut mucosal biopsy[46, 47]. In addition, mechanism of action varies depending on the type of species within one genus. In species level, Bifidobacterium longum showed significant reduction in Sjögren’s syndrome compared to environmental dry eye syndrome (p = 0.004) and controls (p = 0.002), while other species such as Bifidobacterium adolescentis and Bifidobacterium bifidum maintained their normal composition in our study. Given that Bifidobacterium adolescentis and Bifidobacterium longum typically dominates adult gut microbiota[36], further studies are required to investigate their specific interactions and functions in Sjögren’s syndrome at both genus and species levels. Another limitation would be that due to the nature of this study, no additional auto-antibody follow ups were done aside from at the point of disease diagnosis, and so, possible changes in auto-antibodies in our Sjögren’s syndrome subjects were not able to be checked. Relation between auto-antibodies and gut dysbiosis may be an interesting topic to study about in the future. Also, we did not perform any additional lacrimal gland biopsies to confirm current disease activity of Sjögren’s syndrome. Dry eye indices such as tear secretion or unstable tear film status can represent both current disease severity and chronicity. However, without confirmation of the degree of lymphocytic infiltrations, defining current disease activity or chronicity is difficult. Therefore, our study was only able to focus on investigating the relation between current dry eye severity, or signs, and gut dysbiosis. Further investigation between gut dysbiosis and lymphocytic infiltration degree of exocrine glands may provide much information on the pathogenesis of Sjögren’s syndrome. Nevertheless, our study is worthy of notice by 1) providing supporting evidence of gut dysbiosis of human in Sjögren’s syndrome and 2) newly revealing correlation of gut dysbiosis with severity of the dry eye indices. It provides basic information to understand the pathophysiology of gut dysbiosis for future probiotics therapy in Sjögren’s syndrome.
